# Differential responses to group and individual emotional voices: a behavioral and ERP study

**DOI:** 10.1093/scan/nsag041

**Published:** 2026-06-03

**Authors:** Shuqin Liu, Min Shao, Yudie Zhang, Lu Chen, Lu Yu, Rong Huang, Jiayi Shi, Jingyang Yu, Jing Meng

**Affiliations:** Research Center for Brain and Cognitive Science, Chongqing Normal University, Chongqing 401331, China; Key Laboratory of Applied Psychology, Chongqing Normal University, Chongqing 401331, China; Chongqing Key Laboratory of Brain-Inspired Cognitive Computing and Educational Rehabilitation for Children with Special Needs, Chongqing Normal University, Chongqing 401331, China; Research Center for Brain and Cognitive Science, Chongqing Normal University, Chongqing 401331, China; Key Laboratory of Applied Psychology, Chongqing Normal University, Chongqing 401331, China; Chongqing Key Laboratory of Brain-Inspired Cognitive Computing and Educational Rehabilitation for Children with Special Needs, Chongqing Normal University, Chongqing 401331, China; Research Center for Brain and Cognitive Science, Chongqing Normal University, Chongqing 401331, China; Key Laboratory of Applied Psychology, Chongqing Normal University, Chongqing 401331, China; Chongqing Key Laboratory of Brain-Inspired Cognitive Computing and Educational Rehabilitation for Children with Special Needs, Chongqing Normal University, Chongqing 401331, China; Research Center for Brain and Cognitive Science, Chongqing Normal University, Chongqing 401331, China; Key Laboratory of Applied Psychology, Chongqing Normal University, Chongqing 401331, China; Chongqing Key Laboratory of Brain-Inspired Cognitive Computing and Educational Rehabilitation for Children with Special Needs, Chongqing Normal University, Chongqing 401331, China; Research Center for Brain and Cognitive Science, Chongqing Normal University, Chongqing 401331, China; Key Laboratory of Applied Psychology, Chongqing Normal University, Chongqing 401331, China; Chongqing Key Laboratory of Brain-Inspired Cognitive Computing and Educational Rehabilitation for Children with Special Needs, Chongqing Normal University, Chongqing 401331, China; Research Center for Brain and Cognitive Science, Chongqing Normal University, Chongqing 401331, China; Key Laboratory of Applied Psychology, Chongqing Normal University, Chongqing 401331, China; Chongqing Key Laboratory of Brain-Inspired Cognitive Computing and Educational Rehabilitation for Children with Special Needs, Chongqing Normal University, Chongqing 401331, China; Research Center for Brain and Cognitive Science, Chongqing Normal University, Chongqing 401331, China; Key Laboratory of Applied Psychology, Chongqing Normal University, Chongqing 401331, China; Chongqing Key Laboratory of Brain-Inspired Cognitive Computing and Educational Rehabilitation for Children with Special Needs, Chongqing Normal University, Chongqing 401331, China; Research Center for Brain and Cognitive Science, Chongqing Normal University, Chongqing 401331, China; Key Laboratory of Applied Psychology, Chongqing Normal University, Chongqing 401331, China; Chongqing Key Laboratory of Brain-Inspired Cognitive Computing and Educational Rehabilitation for Children with Special Needs, Chongqing Normal University, Chongqing 401331, China; Research Center for Brain and Cognitive Science, Chongqing Normal University, Chongqing 401331, China; Key Laboratory of Applied Psychology, Chongqing Normal University, Chongqing 401331, China; Chongqing Key Laboratory of Brain-Inspired Cognitive Computing and Educational Rehabilitation for Children with Special Needs, Chongqing Normal University, Chongqing 401331, China

**Keywords:** emotion, group empathy, auditory, ERP

## Abstract

Human beings live in social groups, and processing group emotions is crucial for interacting with and understanding others’ behaviors and feelings. This study investigated the cognitive and neural mechanisms of processing group versus individual emotional voices within the auditory modality. We examined ERP responses of 34 participants to group and individual voices across three emotional valences (negative, neutral, positive). Behavioral results revealed a significant interaction: group voices yielded shorter reaction times for neutral and positive emotions but longer for negative ones, alongside more positive overall emotional reactions. Electrophysiologically, group voices elicited more positive N1 and larger P2 amplitudes, with shorter latencies. More specifically, for negative and positive voices, group voices elicited larger P2 amplitudes than individual voices, while no significant difference between group and individual voices emerged for neutral voices. Furthermore, P2 amplitudes elicited by group voices were negatively correlated with participants’ trait cognitive empathy. These results suggest that group voices elicit stronger early neural responses driven by an interplay of acoustic and social salience. Importantly, rather than demonstrating a direct empathy-specific neural mechanism, the observed negative correlation suggests that trait empathy may play a role in modulating the early sensory detection and attentional processing of complex, emotional group empathy.

## Introduction

Emotional expression and perception are an integral part of human interactions (Decety and Jackson 2004, Goldenberg et al. 2020). From an early developmental stage, children demonstrate the capacity to perceive emotions within group contexts (Powell and Spelke 2013). The capacity to understand the emotions of an individual or a group is essential for successful social interactions (Singer et al. 2004, Smith et al. 2007). Furthermore, the ability to perceive and assess the emotions of a group may be more evolutionarily significant than processing individual emotions. In everyday experiences, many emotions arise in group settings or during group events (van Kleef and Fischer 2016), where individuals often navigate scenarios in which multiple people simultaneously express emotions. Consequently, efficient processing of group emotional signals may improve an individual’s capacity to recognize collective emotional states, thereby promoting social integration. Despite this, the existing emotion research predominantly emphasizes individual emotion, with comparatively less focus on the processing of group emotional signals. To address this gap, this study was designed to systematically investigate the distinctions between neural responses to group-based and individual emotional voices, with a specific aim to uncover the spatiotemporal neural dynamics underlying their processing within the auditory modality.

In everyday life, people are confronted with an abundance of different emotional stimuli from the environment. Typically, these cues are transmitted through multiple sensory channels, with audiovisual stimuli being particularly prevalent (Gerdes et al. 2013). It is worth noting that while visual cues have been extensively studied, the auditory modality represents an equally, if not more, critical channel for social signaling (Schirmer and Kotz 2006, Belin et al. 2008). In real-world social contexts, emotional signals often manifest as collective vocalizations—such as the synchronized cheering of a crowd or the shared wailing of grief—rather than isolated individual voices (van Kleef and Fischer 2016). These group voices possess unique acoustic signatures and social salience that better simulate naturalistic environments, yet the neural mechanisms underlying the processing of such collective auditory signals remain largely unexplored.

Previous studies on perception in group emotion contexts have largely concentrated on the influence of ambient group emotional information on the reactions toward a target individual (Gray et al. 2017, Cao et al. 2023, Wang et al. 2024b). In a previous study (Mumenthaler et al. 2018), participants were presented with a target face alongside a surrounding face displaying a different emotion. The results indicated that when the emotional expressions of the target and surrounding faces were congruent, participants responded more quickly and accurately in identifying the target face’s emotion compared to conditions where the emotional expressions were incongruent (Mumenthaler et al. 2018). Similarly, it was found that context directly regulates emotional judgments, for instance, by reinforcing attention to specific faces through scene cues (Hao et al. 2025).

In the field of empathy for pain research, a previous study (Mei et al. 2024) using event-related potential (ERP) techniques in the visual modality explored 2 × 2 arrays of faces in which 4 painful faces, 1 painful + 3 non-painful faces, or 4 non-painful faces were presented. They found that compared to 1 painful + 3 non-painful faces, 4 painful faces elicited enhanced empathic responses, including higher pain intensity scores, more negative emotional reactions, and larger P2 amplitudes. Regarding the auditory modality, a recent study investigated differential neural processing of multi-voice versus single-voice stimuli using ERPs (Shao et al. 2024b). By comparing responses to group painful voices and individual painful voices, the results revealed that group signals elicited larger P2 amplitudes and stronger behavioral responses than individual signals, suggesting that stimuli involving multiple overlapping voices trigger enhanced early neural reactivity and arousal in the auditory modality. Nevertheless, existing literature lacks evidence on the processing of group vocalizations for basic emotions in the auditory modality. Unlike pain, which signals immediate physical threat, basic emotions such as joy or sadness play crucial roles in social bonding and affiliation; thus, it remains to be determined whether the group enhancement effect generalizes to these distinct social signals.

Beyond the social content typically associated with group interactions, crucial recent research highlights that empathy is not solely driven by social factors but is significantly modulated by spatial and perceptual cues. For instance, previous studies have demonstrated that the magnitude of empathic ERP responses to pain is modulated by perceived physical distance, with closer stimuli eliciting stronger neural reactions due to their higher threatening value (Schiano Lomoriello et al. 2018). Similarly, the possibility of sensorimotor interaction—mediated by peripersonal space—is essential for triggering full empathic responses; when interaction is prevented, both early and late empathic components are significantly reduced (Schiano Lomoriello et al. 2023). Furthermore, the temporal dynamics of empathy depend critically on the type of perceptual cue available, with early components being highly sensitive to low-level sensory features (Meconi et al. 2018). These findings suggest that the “group” effect in empathy might not arise solely from social appraisal but could also stem from the increased acoustic richness and spatial salience of collective vocalizations. Investigating group vocalizations presents a unique challenge: the digital superposition of multiple voices inevitably increases spectral complexity and energy density. Therefore, a central question of the present study is whether the observed neural differences reflect a specialized response to social group signals, a generalized physiological response to acoustic richness, or a nuanced interplay between the two.

To address the aforementioned issues, the present study aimed to produce group emotional (positive, neutral, and negative) voices and utilize ERP techniques to investigate the behavioral and electrophysiological dynamics of processing group versus individual emotional voices. Specifically, we focused on early components like P2, which are sensitive to both acoustic features and top-down social attention, making them ideal for exploring the interplay between physical and social salience. Based on previous studies (Sheng and Han 2012, Sheng et al. 2014, Meng et al. 2019a, Mei et al. 2024, Shao et al. 2024a), we hypothesized that group voices would elicit differential behavioral and ERP responses compared to individual voices. Crucially, we aimed to explore whether this enhancement is purely driven by the higher acoustic complexity inherent in superimposed vocalizations, or if it reflects an interactive process involving the biological and social significance of collective emotional signals.

## Methods

### Participants

Participants were recruited by means of posters and online advertising in Chongqing Normal University, Chongqing, China. An a priori power analysis was conducted using G*Power 3. The analysis indicated that for a 2 × 3 within-subject design (assuming ɑ = 0.05 and a correlation among repeated measures of 0.5), a minimum of 19 participants would be required to detect a medium effect size (Cohen’s *f *= 0.25) with 0.80 power. This study involved 34 participants (*Mean _age_* = 20.44 years, SD _*age*_ = 1.52 years, range = 18–23 years, 16 females). They reported no history of mental illness, had normal or corrected-to-normal vision, and identified as right-handed. Additionally, participants confirmed via self-report that they possessed normal hearing acuity and had no history of hearing disorders. Regarding musical background, none of the participants were professional musicians or had undergone formal musical training (e.g. extensive instrumental lessons for > 2 years). Prior to commencing the experiment, all participants received comprehensive written and verbal disclosures detailing the study’s objectives, procedures, potential risks/benefits, and their right to withdraw at any time without penalty. Following confirmation of their full comprehension, each participant voluntarily provided written informed consent by signing a designated consent form, which formally confirmed their agreement to participate in the research. The study was approved by the Academic Ethics Committee of School of Educational Sciences, Chongqing Normal University, and all procedures were performed in accordance with ethical guidelines and regulations.

### Stimuli

The individual emotional voices employed in this study were adapted from the Montreal Affective Voices database (Belin et al. 2008), which depicted human emotional voices of interjections, such as anger, sadness, happiness, and surprise. We selected 10 individual negative (sadness) voices, 10 individual neutral voices, and 10 individual positive (happiness) voices (50% female voices). All auditory processing and synthesis were conducted using Adobe Audition 2022 software (Adobe Systems Inc.).

**Figure 1:**
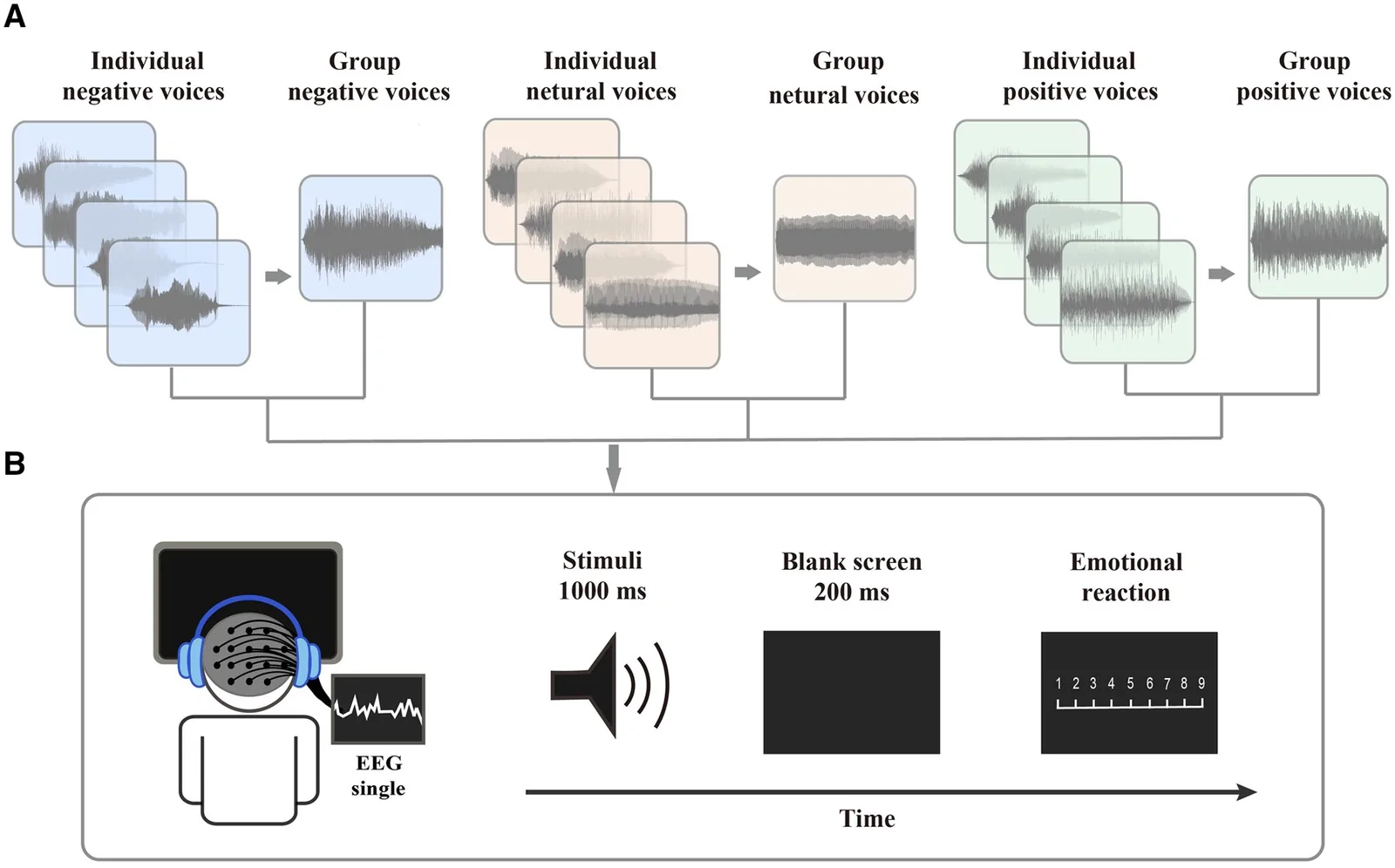
Flowchart describing the stimuli examples, experimental design and procedure. (A) The examples of waveforms of group and individual negative, neutral, and positive voices. Each group negative, neutral, and positive voice was made by superimposing 4 randomly selected individual negative, neutral, and positive voices, respectively. (B) Experimental design and procedure. EEG signals were simultaneously recorded while participants performed the experimental task.

Based on the auditory stimulus generation protocol from a previous study (Shao et al. 2024b), 10 unique group voice combinations were pre-generated for each emotional category. Each combination consisted of 4 distinct individual voices (50% female voices) selected from the respective pool, as illustrated in Fig. 1A. This mixed-gender composition was employed to create a specific type of additive group signal. While it captures an increased social density compared to individual voices, we acknowledge it acts as a controlled, superimposed multi-voice signal rather than a fully naturalistic collective vocalization. During synthesis, the 4 individual voices were digitally superimposed using strict onset alignment, with all waveforms starting simultaneously at the 0 ms mark. No random temporal jitter was introduced between the voices to ensure that the ERP triggering was time-locked to the synchronized onset of all vocal signals, thereby preserving the temporal precision of early ERP components such as N1 and P2. Crucially, these 10 fixed group combinations per condition were presented consistently to all participants to maximize experimental control.

Finally, based on previous research (Yang et al. 2022b, Huo et al. 2025) and to ensure temporal consistency for ERP analysis, all individual and group clips were standardized to a duration of 1000 ms. Specifically, shorter clips were padded with silence at the end, while longer clips were truncated at the 1000 ms mark. Importantly, no time-stretching or compression techniques were applied, thereby preserving the original pitch and emotional prosody of the vocalizations. To conclude the preparation, the root-mean-square intensity of all stimuli was normalized to 70 dB, ensuring loudness consistency across all experimental trials.

### Measurement of trait cognitive and affective empathy

The trait cognitive empathy and trait affective empathy of participants were measured using the Questionnaire of Cognitive and Affective Empathy (Reniers et al. 2011). This scale consists of a total of 31 items. Participants responded to each item using a 4-point Likert scale (1 = *strongly disagree*, 2 = *slightly disagree*, 3 = *slightly agree*, 4 = *strongly agree*). The scale encompasses 5 dimensions: perspective taking, online simulation, emotional contagion, proximal responsivity, and peripheral responsivity. Specifically, the perspective taking and online simulation dimensions assessed trait cognitive empathy, while emotional contagion, proximal responsivity, and peripheral responsivity dimensions assessed trait affective empathy. Psychometric evaluations confirmed that the Chinese version of the Questionnaire of Cognitive and Affective Empathy exhibited both good reliability and validity (Liang et al. 2019). In this study, the Cronbach’s α coefficient was 0.79.

### Procedure

To ensure optimal data quality and participant comfort, the experimental sessions were conducted in a dedicated, sound-attenuated chamber, which was maintained at a constant thermoneutral temperature of approximately 22 °C. Prior to the commencement of the experiment, all participants completed the Questionnaire of Cognitive and Affective Empathy. Stimuli were presented using the E-Prime (3.0) program (Psychology Software Tools, Inc, Pittsburgh, PA, USA). Initially, a 1000 ms voice was presented randomly. Participants were required to press a specific key (either “1,” “2, or “3”) as quickly and accurately as possible to judge the emotional valence of the voice (negative, neutral, or positive). Key-pressing was counterbalanced across participants to control for order effect. After judging the emotional valence, 200 ms later, participants were instructed to rate their subjective emotional reactions to each voice, based on a 9-point Likert scale (1 = *very unhappy*, 5 = *neutral*, 9 = *very happy*). A random inter-trial interval of 1000–2000 ms occurred between trials (refer to Fig. 1B for detailed procedure information). Each emotional voice stimulus was repeated 4 times in a pseudo-randomized sequence, culminating in 240 total trials.

### Electroencephalography recording

EEG data were recorded using a 32-electrode cap with tin electrodes mounted on an actiCHamp system (Brain Vision LLC, Morrisville, NC, USA). The electrode on the average of the mastoids was used as the recording reference, and the medial frontal aspect was used as a ground electrode. All electrode impedances remained below 5 kΩ.

### Electroencephalography data analysis

EEG data were preprocessed and analyzed via MATLAB R2016a (MathWorks, Natick, MA, USA) and the EEGLAB toolbox (Delorme and Makeig 2004). Continuous EEG signals were band-passed, filtered (0.1–40 Hz), and segmented using a 1200 ms time window. Time windows of 200 ms before and 1000 ms after the onset of voice stimuli were extracted from the continuous EEG. EEG epochs were baseline-corrected by a 200 ms time interval prior to voice stimuli onset. Electro-oculographic artifacts were corrected with an independent component analysis algorithm (Jung et al. 2001).

A priori based on previous studies (Fan et al. 2014, Cui et al. 2016, Peng et al. 2019, Meng et al. 2020, Shao et al. 2024a), and after visually confirming the peak latencies and scalp topographies in both the single-participant and group-level grand average ERP waveforms, dominant ERP components were identified as follows: N1 (Fz, Cz, FC1, FC2), P2 (Fz, Cz, FC1, FC2), and LNC (Fz, F3, F4, FC1, FC2). Amplitudes of N1 and P2 were both measured at the frontal-central electrodes and calculated as the average ERP amplitudes within N1 latency intervals of 132–192 ms and P2 latency intervals of 212–272 ms. Amplitudes of LNC were measured at the prefrontal electrodes and calculated as the average ERP amplitudes within latency intervals of 400–800 ms.

### Statistical analyses

Data analyses were performed using SPSS 15.0 software (IBM Corp., Armonk, NY, USA). Behavioral data (accuracy, reaction time, emotional reaction) and ERP data (N1, P2, and LNC amplitude and latency) were analyzed using a repeated-measures analysis of variance (ANOVA) of 2 “stimulus type” (individual voice, group voice) × 3 “emotion type” (negative, neutral, positive). If the interactions between the 2 factors were significant, simple effect analysis was performed between 2 stimulus types for each condition and reported in the results. We corrected the degrees of freedom of the *F*-value using the Greenhouse–Geisser test.

Furthermore, to mitigate the influence of differences in physical properties (such as frequency and timbre) between group and individual voices on the ERP results, the present study also calculated differential ERP amplitudes between group and individual voices for negative, neutral, and positive voices. Additionally, repeated measures ANOVA were employed to compare the differential ERP amplitudes between negative, neutral, and positive voices. If the main effect was significant, we performed post-hoc tests.

Finally, we investigated the relationships among trait cognitive empathy, trait affective empathy, and ERP amplitudes using Pearson’s correlation. The correlation analysis was corrected for false discovery rate (FDR) to control Type I error. Specifically, the Benjamini–Hochberg method was used to adjust the *P* value obtained from Pearson correlation tests.

## Results

### Behavioral results

#### Accuracy

The main effect of “stimulus type” was significant (*F*  _(1, 33)_ = 31.13, *P *< .001, η^2^_p_ = 0.49), participants showed higher accuracies in distinguishing individual voices (0.95 ± 0.01) than group voices (0.89 ± 0.02). The main effect of “emotion type” was significant (*F*  _(2, 66)_ = 14.70, *P *< .001, η^2^_p_ = 0.31), accuracies in judging negative voices (0.85 ± 0.02) were lower than neutral (0.95 ± 0.02; *P *= .001) and positive (0.97 ± 0.01; *P *< .001) voices, but there was no significant difference between neutral and positive voices (*P *= .339). The interaction effect between “stimulus type” and “emotion type” was significant (*F*  _(1.29, 42.56)_ = 22.72, *P *< .001, η^2^_p_ = 0.41). Simple effect analysis revealed that, for the negative voices, lower accuracies were found in group negative voices (0.78 ± 0.03) than individual negative voices (0.93 ± 0.01; *P *< .001), whereas no significant difference between group and individual voices was found for neutral (*P *= .118) and positive (*P *= .070) voices.

#### Reaction time

The main effect of “emotion type” was significant (*F*  _(2, 66)_ = 64.23, *P *< .001, η^2^_p_ = 0.66), participants responded faster to neutral voices (1072.86 ms ± 31.29 ms) than negative voices (1286.57 ms ± 32.57 ms; *P *< .001) and positive voices (1167.53 ms ± 30.21 ms; *P *< .001); participants responded faster to positive voices than negative voices (*P *< .001). The interaction effect between “stimulus type” × “emotion type” was significant (*F*  _(1.69, 55.66)_ = 13.68, *P *< .001, η^2^_p_ = 0.29). Simple effect analysis revealed that for negative voices, participants responded slower to group negative voices (1313.64 ms ± 37.21 ms) than individual negative voices (1259.50 ms ± 30.56 ms; *P *= .008), while for neutral and positive voices, participants responded faster to group voices (group neutral voices: 1046.52 ms ± 32.65 ms; group positive voices: 1144.83 ms ± 33.88 ms) than individual voices (individual neutral voices: 1099.20 ms ± 32.56 ms, *P *= .007; individual positive voices: 1190.23 ms ± 27.72 ms, *P *= .002).

#### Emotional reaction

The main effect of “stimulus type” (*F*  _(1, 33)_ = 23.52, *P *< .001, η^2^_p_ = 0.42) was significant, participants exhibited more positive emotional reactions to group voices (5.14 ± 0.10) than individual voices (4.79 ± 0.07). The main effect of “emotion type” was significant (*F*  _(2, 66)_ = 107.45, *P *< .001, η^2^_p_ = 0.77). In contrast to negative voices (3.17 ± 0.20), participants reacted more positively to neutral voices (4.41 ± 0.17; *P *< .001) and positive voices (7.31 ± 0.19; *P *< .001); positive voices were judged more positively than neutral voices (*P *< .001). The interaction effect of “stimulus type” × “emotion type” was also significant (*F*  _(1.55, 51.18)_ = 23.70, *P *< .001, η^2^_p_ = 0.42). Simple effect analysis revealed that for negative voices, participants exhibited more positive emotional reactions to group negative voices (3.65 ± 0.23) than individual negative voices (2.69 ± 0.19; *P *< .001), while no significant difference between individual and group neutral voices (*P *= .908) and positive voices (*P *= .270) was found.

No other significant main effects or interactions were found (all *P*s > .05). For more details of the behavioral results, please see the Supplementary Material.

### ERP results

#### ERP amplitudes

**Figure 2:**
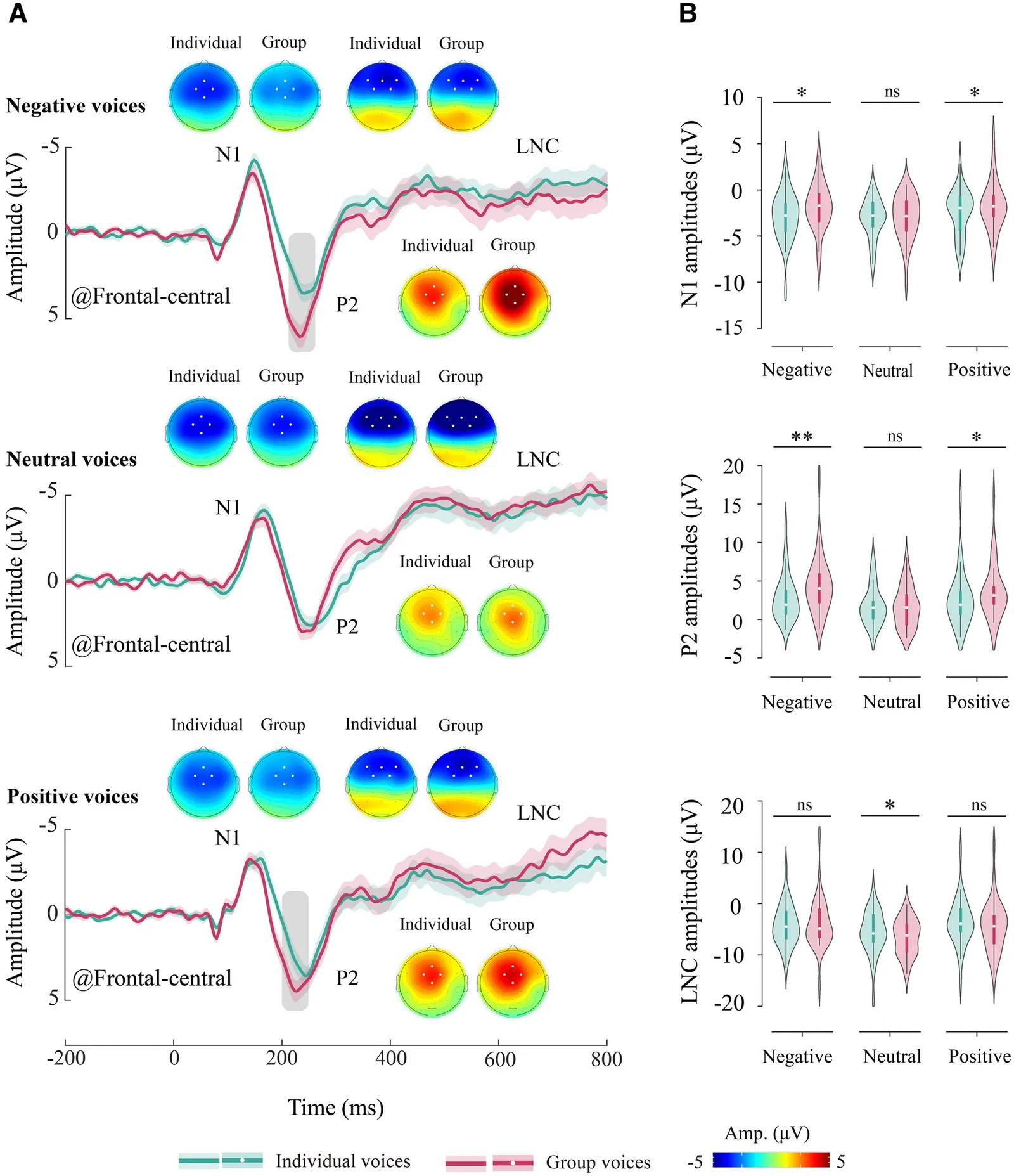
ERP results for different types of voices. (A) ERP waveforms and scalp topography distributions elicited by different types of voices, including negative group and individual voices (top panel), neutral group and individual voices (middle panel), and positive group and individual voices (bottom panel). Solid lines show the average waveforms. Shaded areas represent the ±1 standard error of the mean (*SEM*). The white dots in the scalp topographies indicate the electrodes included in the analysis. (B) Violin plots depict the N1, P2, and LNC amplitudes. Box plots illustrate the quartile range of the data, with the white dot representing the median. *P < 0.05, **P < 0.01.

**Figure 3:**
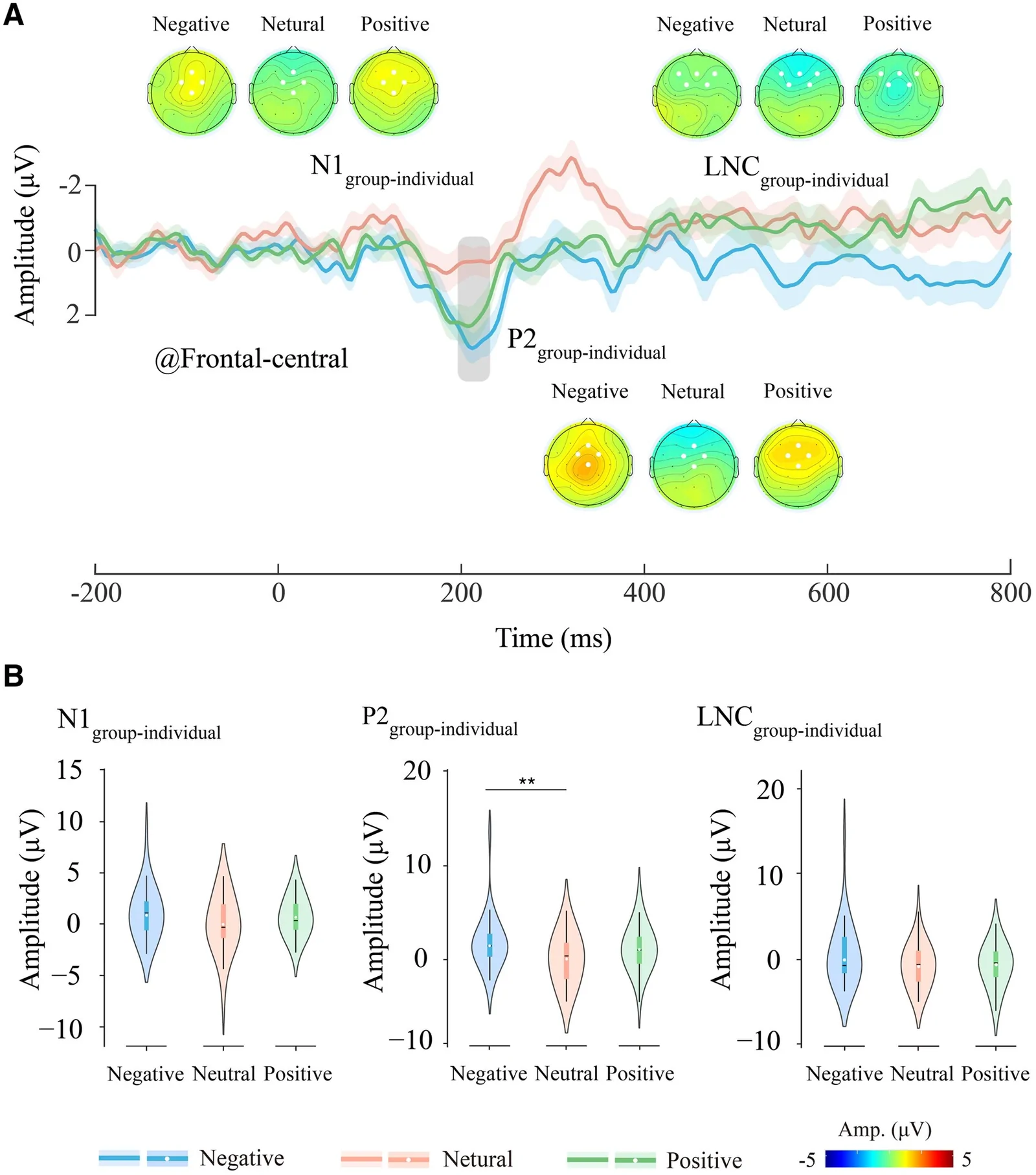
The differential ERP waveforms of group and individual voices. (A) Waveform and topographical maps of differential ERP of negative (blue), neutral (red), and positive (green) voices. Solid lines show the average waveforms. Shaded areas represent the ±1 *SEM*. The regions marked by white dots in the topographical maps indicate the electrode points included in the analysis. (B) Results of difference tests of the differential ERP amplitudes between group and individual voices. Violin plots illustrate the distribution of the data, while box plots depict the quartile range of the data, with white dots representing the median. **P < 0.01.

ERP waveforms, scalp topographies, and bar charts are shown in Fig. 2. Results of the statistical analyses of the ERP amplitudes are summarized in Table 1. Differential ERP waveforms and scalp topography distributions are presented in Fig. 3.

**Table 1:** Results of the statistical analyses of ERP amplitudes.

Variable	N1			P2			LNC		
	*F*	*P*	η^2^_p_	*F*	*P*	η^2^_p_	*F*	*P*	η^2^_p_
Stimulus type	**6.94**	**0.013**	**0.17**	**13.05**	**0.001**	**0.28**	2.38	0.133	0.07
Emotion type	**5.02**	**0.009**	**0.13**	**16.27**	**<0.001**	**0.33**	**14.25**	**<0.001**	**0.30**
Stimulus type × Emotion type	1.21	0.305	0.04	**4.11**	**0.021**	**0.11**	2.00	0.143	0.06

*Note.* The results were obtained through repeated measures analysis of variance, including “stimulus type” (individual voice, group voice) and “emotion type” (negative, neutral, positive). Significant effects (*P *< 0.05) were highlighted in bold.

**N1.** The main effect of “stimulus type” (*F*  _(1, 33)_ = 6.94, *P *= .013, η^2^_p_ = 0.17) was significant, group voices (−2.19 μV ± 0.37 μV) elicited more positive N1 amplitudes than individual voices (−2.81 μV ± 0.33 μV). The main effect of “emotion type” (*F*  _(2, 66)_ = 5.02, *P *= .009, η^2^_p_ = 0.13) was significant, positive voices (−2.09 μV ± 0.41 μV) elicited more positive N1 amplitudes than neutral voices (−3.07 μV ± 0.32 μV; *P *= .021), negative voices (−2.34 μV ± 0.39 μV) elicited marginally more positive N1 amplitudes than neutral voices (*P *= .051). However, no significant difference was found between positive and negative voices (*P *= .460).

**P2.** The main effect of “stimulus type” was significant (*F*  _(1, 33)_ = 13.05, *P *= .001, η^2^_p_ = 0.28), group voices (3.24 μV ± 0.50 μV) elicited larger P2 amplitudes than individual voices (2.36 μV ± 0.45 μV). The main effect of “emotion type” was significant (*F*  _(2, 66)_ = 16.27, *P *< .001, η^2^_p_ = 0.33), negative (3.63 μV ± 0.53 μV; *P *< .001) and positive (3.09 μV ± 0.58 μV; *P *= .005) voices elicited larger P2 amplitudes than neutral voices (1.68 μV ± 0.39 μV). However, no significant difference was observed between the negative and positive voices (*P *= .268). The interaction effect between “stimulus type” and “emotion type” was significant (*F*  _(2, 66)_ = 4.11, *P *= .021, η^2^_p_ = 0.11). Simple effect analysis indicated that compared to individual negative voices (2.79 μV ± 0.51 μV) and individual positive voices (2.57 μV ± 0.61 μV), group negative voices (4.47 μV ± 0.66 μV; *P *= .002) and group positive voices (3.61 μV ± 0.62 μV; *P *= .010) elicited larger P2 amplitudes, respectively, whereas no difference between individual and group neutral voices was found (1.74 μV ± 0.44 μV vs. 1.63 μV ± 0.46 μV; *P *= .802).

**LNC.** The main effect of “emotion type” was significant (*F*  _(2, 66)_ = 14.25, *P *< .001, η^2^_p_ = 0.30), negative (−4.08 μV ± 0.70 μV; *P *< .001) and positive (−2.09 μV ± 0.41 μV; *P *< .001) voices elicited less negative LNC amplitudes than neutral voices (−6.11 μV ± 0.63 μV). However, no significant difference was found between negative and positive voices (*P *= .989).

**Differential ERP amplitudes.** Differential ERP waveforms and scalp topography distributions are presented in Fig. 3A, and the violin plots of differential ERP amplitudes are shown in Fig. 3B. The main effect of “emotion type” of P2_group–individual_ amplitudes was significant (*F*  _(2, 66)_ = 4.11, *P *= .021, η^2^_p_ = 0.11), negative voices (1.68 μV ± 2.89 μV) elicited larger P2_group–individual_ amplitudes than neutral voices (−0.11 μV ± 2.49 μV; *t*_(33)_ = 2.81, *P *= .008, Cohen’s *d *= 0.66), positive voices (1.04 μV ± 2.22 μV) elicited marginally larger P2_group–individual_ amplitudes than neutral voices (*t*_(33)_ = −1.99, *P *= .055, Cohen’s *d *= 0.49). However, no significant difference was found between negative and positive voices (*P *= .347). No other significant main effect was found (all *P*s > .05).

#### ERP latencies

**N1 latencies.** The main effect of “stimulus type” was significant (*F*  _(1, 33)_ = 40.00, *P *< .001, η^2^_p_ = 0.55), group voices (152.44 ms ± 1.60 ms) elicited shorter N1 latencies than individual voices (158.88 ms ± 1.72 ms). The main effect of “emotion type” was significant (*F*  _(2, 66)_ = 41.58, *P *< .001, η^2^_p_ = 0.56), neutral voices (162.96 ms ± 1.64 ms) elicited longer N1 latencies than negative voices (149.41 ms ± 1.44 ms) and positive voices (154.62 ms ± 2.23 ms). No other significant effect was found (all *P*s > .05).

**P2 latencies.** The main effect of “stimulus type” was significant (*F*  _(1, 33)_ = 27.23, *P *< .001, η^2^_p_ = 0.45), group voices (237.84 ms ± 1.90 ms) elicited shorter P2 latencies than individual voices (245.75 ms ± 1.68 ms). The main effect of “emotion type” was significant (*F*  _(2, 66)_ = 19.05, *P *< .001, η^2^_p_ = 0.37), neutral voices (249.00 ms ± 2.27 ms) elicited longer P2 latencies than negative voices (240.04 ms ± 1.71 ms) and positive voices (236.34 ms ± 2.07 ms). No other significant effect was found (all *P*s > .05).

### Correlation analysis

**Figure 4:**
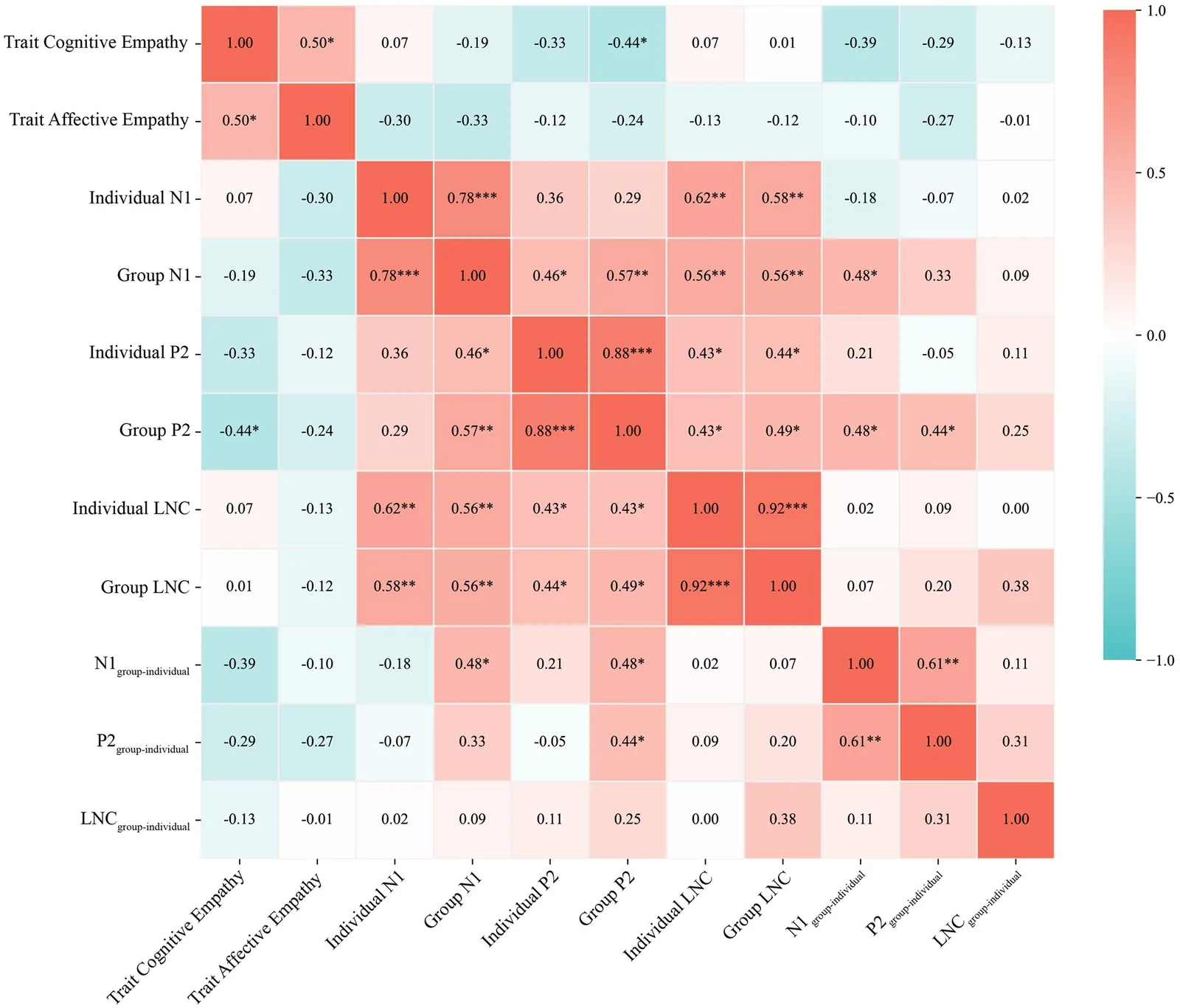
Correlation between trait empathy (trait cognitive empathy and trait affective empathy) and ERP (group N1, P2, and LNC; individual N1, P2, and LNC; N1_group–individual_, P2_group–individual_, and LNC_group–individual_) amplitudes. *P < 0.05, **P < 0.01, ***P < 0.001 (FDR-corrected).

Correlations between the ERP amplitudes and trait empathy are shown in Fig. 4. Pearson correlation results showed that participants’ trait cognitive empathy was negatively correlated with group P2 amplitudes (*r *= −0.44, *P *= .032, FDR-corrected). However, further analysis using the differential ERP amplitudes revealed that the correlation between trait cognitive empathy and the P2_group–individual_ difference wave did not reach statistical significance (*r *= −0.29, *P *= .189, FDR-corrected).

## Discussion

To investigate the characteristics and neural mechanisms of processing group emotions in the auditory domain, the present research combined behavioral methodology with EEG techniques. The behavioral results demonstrated a significant interaction between stimulus type and emotion type: while participants exhibited shorter reaction times to group neutral and positive voices compared to individual voices, they showed longer reaction times to group negative voices. Furthermore, participants exhibited more positive overall emotional reactions to group voices. The ERP results demonstrated that participants had more positive N1 and larger P2 amplitudes for group voices than for individual voices. More specifically, for negative and positive voices, group voices elicited larger P2 amplitudes than individual voices, while no significant difference between group and individual voices emerged for neutral voices. These findings demonstrate distinct neural processing patterns for group emotions and suggest that neural responses to group voices are stronger than those to individual voices in the auditory modality. Importantly, the enhanced P2 for group emotional voices indicates a heightened detection of salient auditory signals. This finding, combined with the lack of group enhancement in the neutral condition, provides a more nuanced picture of how the brain balances physical input and social meaning, suggesting that this enhancement arises from a complex interplay between low-level acoustic complexity and higher-order social-emotional integration.

For neutral and positive stimuli, participants exhibited shorter reaction times in identifying group voices. This rapid processing is potentially due to the conspicuous survival importance of group-related cues (Haberman and Whitney 2007), which, in turn, facilitated enhanced attentional allocation to affiliative emotional cues. This aligns with previous theoretical perspectives (Nakahashi and Ohtsuki 2018) that group-living animals evolve mechanisms for collective emotion processing to balance individual sensitivity and group efficiency, prioritizing the rapid detection of affiliative signals to promote social integration.

Regarding the behavioral results for negative voices, participants exhibited lower accuracy, longer reaction times, and less negative subjective ratings for group negative voices compared to individual voices. Rather than viewing these results as contradictory, this behavioral dissociation can be best explained by a sequential process initiated by acoustic masking. When four distinct negative vocalizations are superimposed, the resulting spectral overlap creates a “perceptual blurring” effect (Goldenberg et al. 2020) that makes it inherently difficult for listeners to isolate specific emotional cues, directly leading to the observed categorization uncertainty (i.e. lower accuracy and longer RTs). Crucially, this low-level perceptual ambiguity subsequently influences high-level subjective evaluations: when participants are uncertain about the severity of a negative signal due to acoustic masking, they tend to adopt a conservative rating strategy, resulting in a downstream positivity bias driven by uncertainty rather than a genuine reduction in negative perception. Interestingly, this behavioral degradation was accompanied by an enhanced P2 amplitude. Because the P2 component is sensitive to acoustic complexity and attention allocation (Lijffijt et al. 2009), the enlarged P2 for group-negative voices likely does not reflect a stronger perception of negative emotion, but rather an increased neural mobilization triggered by stimulus ambiguity, acting as a compensatory mechanism deployed to decode the acoustically masked signals.

The N1 component is primarily associated with early auditory sensory processing and acoustic feature detection (Choi et al. 2014). In the present study, group voices elicited more positive N1 amplitudes than individual voices. Given that the superposition of multiple voices inevitably increases the acoustic density and spectral overlap compared to single voices, this reduced N1 amplitude likely reflects an early sensory gating or suppression mechanism. When confronted with highly complex and acoustically dense signals, the auditory system may automatically attenuate early sensory encoding to prevent sensory overload and filter out competing acoustic noise (Lijffijt et al. 2009). This early sensory suppression of physical noise then serves as a prerequisite for the subsequent heightened neural mobilization (indexed by the larger P2) deployed to decode the social-emotional meaning.

Similarly, the P2 component is known to be highly sensitive to the spectral complexity, intensity, and arousal levels of auditory stimuli (Lijffijt et al. 2009, Schiano Lomoriello et al. 2018). Our finding of larger P2 amplitudes for group voices likely reflects the neural response to the increased acoustic complexity and energy inherent in superimposed vocalizations. Rather than directly representing “emotional contagion,” this enhanced P2 suggests that group voices function as high-arousal stimuli that trigger significant neural mobilization. This interpretation aligns with the view that the brain prioritizes the processing of complex, high-intensity social signals (Schiano Lomoriello et al. 2018). While speculative, this heightened sensory and physiological arousal (indexed by P2) may facilitate the rapid detection of potential environmental threats or social cues, thereby supporting an adaptive “social warning” function. Consistent with this interpretation, the analysis of the P2_group–individual_ difference waves revealed that this neural enhancement was statistically robust for negative voices (compared to neutral ones). While the difference between negative and positive voices did not reach significance, this reliable enhancement for negative stimuli suggests that collective distress signals possess high biological salience due to their acoustic roughness and intensity. While we must be cautious not to over-interpret these sensory components as specific empathy mechanisms, the robust neural response to group negative signals likely serves an adaptive role: facilitating the rapid detection of potential shared dangers and alerting the organism to survival threats (de Waal 2008).

A potential concern in studies involving superimposed stimuli is the “acoustic confound,” as combining multiple voices inherently increases spectral complexity and energy density. It could be argued that the larger P2 observed for group voices simply reflects the brain’s response to more complex auditory inputs. However, our findings provide strong evidence against this purely acoustic interpretation. Specifically, if the P2 enhancement were solely driven by physical superposition, we would expect to see similar amplitude increases for neutral group voices. The fact that group enhancement was observed exclusively for emotional (positive and negative) but not neutral voices suggests that the P2 component reflects more than just acoustic complexity or general attentional allocation. Consistent with auditory ERP evidence indicating that the P200 indexes the extraction of emotional salience to facilitate rough stimulus appraisal (Liu et al. 2012), as well as cross-modal findings from the visual domain (Carretié et al. 2004), this selective enhancement suggests that the auditory P2 similarly reflects rapid, arousal-based detection of collective emotional salience. The auditory system appears to rapidly decode the heightened arousal and biological urgency embedded in collective emotional signals. Consequently, the enhanced P2 for emotional group voices represents a rapid, arousal-driven appraisal mechanism that immediately prioritizes highly arousing and socially significant collective signals for deeper cognitive evaluation.

Interestingly, in this study, the P2 amplitudes to group voices exhibited a negative correlation with trait cognitive empathy scores. However, the P2_group–individual_ difference wave revealed that this association was not statistically significant. This suggests that the unique variance attributed to the group condition is limited, and the initial correlation was largely driven by individual differences in baseline neural reactivity. While previous studies link high empathy traits to advantages in emotion recognition (Besel and Yuille 2010, Yang et al. 2022a), relying on a single questionnaire measure is insufficient to establish an empathy-specific neural mechanism here. Because the P2 component is highly sensitive to arousal, acoustic features, and top-down attention (Lijffijt et al. 2009), this observed correlation more likely reflects individual differences in general early sensory gating or attentional filtering. High-empathy individuals may possess more efficient attentional allocation to prevent sensory overload when processing complex, multi-voice social signals, rather than reflecting a direct empathy-specific neural response. Therefore, this finding should be regarded as a preliminary association that warrants verification in future studies with strictly controlled designs.

The LNC amplitude corresponds to the subsequent processing and evaluation phases of emotional information (Meng et al. 2019a, 2019b, Wang et al. 2025). The results of the present study revealed that participants exhibited less negative LNC amplitudes to negative and positive voices than to neutral voices, indicating that late processing requires more cognitive resources for emotion-situational integration. Importantly, however, the stimulus type (group vs. individual) did not substantially impact the LNC amplitudes. This pattern highlights a distinct functional dissociation between early and late neural processing stages. While early sensory components (N1, P2) are highly sensitive to the acoustic richness and social salience of group signals—reflecting automatic bottom-up detection and attentional mobilization—the later stage of cognitive evaluation (indexed by the LNC) appears to be driven primarily by the emotional valence of the stimulus rather than its social density.

### Limitations

Despite the methodological rigor with which these studies were conducted, several limitations should be acknowledged. Firstly, the ERP technique used in this study did not provide evidence regarding the area of brain activation, which may be further explored in future studies using fMRI. Secondly, a critical limitation of the present study concerns the stimulus construction. Group voices were generated by superimposing four individual voices, which introduced differences in spectral complexity and density compared to single voices. Although we matched the average loudness, we did not employ “scrambled” controls to fully isolate the social content from these low-level acoustic features. Consequently, the enhanced early ERP components observed for group voices may be largely driven by these physical differences rather than the social nature of the stimulus. To disentangle sensory processing from genuine social affective processing, future studies should include control stimuli, such as scrambled voices that preserve spectral properties while removing social meaning, to further isolate the contribution of pure acoustic complexity. Thirdly, the ecological validity of our stimuli should be considered with caution. In the present study, group stimuli were created by digitally superimposing four individual voices with onset alignment. While this method controls for total duration and initial onset, real-world group vocalizations (e.g. collective chanting, cheering, or grieving) often involve intricate temporal coordination (rhythmic synchronization) and spatial cues (sound sources from different physical locations). The simple digital overlay used here produces stimuli that are distinct from natural group vocalizations, potentially introducing different acoustic signatures—such as random phase alignments and specific interference patterns. Therefore, our findings specifically reflect the neural processing of “summed” social signals and may not fully generalize to highly coordinated, naturalistic collective vocalizations. Fourthly, although ERP technology provides high time-resolution data on the neural mechanisms of emotional processing, existing research is still challenged by the lack of deep coupling with brain cognitive mechanisms. Future research could incorporate machine learning (Chen 2023), advanced artificial intelligence algorithms (Wang et al. 2024a, Kuai et al. 2025), or multivariate EEG analysis techniques (Li et al. 2024, Zheng and Weng 2025), building a social cognitive engine for social emotion processing through multimodal data fusion to further explore its mechanisms.

## Conclusion

This study utilized individual and group emotional voices as stimuli to investigate the characteristics of behavioral and neural responses toward different categories. The behavioral results revealed a divergent pattern based on emotion type: compared to individual voices, group voices elicited shorter reaction times for neutral and positive emotions, but longer reaction times for negative emotions. Overall, group voices also elicited more positive subjective emotional reactions. Regarding ERP results, group voices elicited stronger early neural responses than individual voices, suggesting heightened sensory and affective processing. Although likely driven in part by acoustic salience, these enhanced responses may facilitate rapid attentional capture and subsequent affective processing in the auditory modality. Overall, this study revealed significant differences in neural and behavioral responses based on voice type (individual vs. group). The significance of these findings is two fold. Firstly, they provide a more granular understanding of the neural mechanisms underlying the processing of social auditory signals, highlighting the brain’s sensitivity to both the acoustic richness and the emotional relevance of collective vocalizations. Secondly, they establish a robust empirical foundation for subsequent applied research, offering insights into how complex, naturalistic social environments modulate early human attention and sensory gating, and providing a valuable framework for understanding the neural basis of group empathy in collective social contexts.

## Supplementary Material

nsag041_Supplementary_Data

## Data Availability

Data associated with this article can be found in the online version at https://pan.baidu.com/s/1LiSEprENf0wqCSt2Nk0fag? pwd=qp9n.
