# Supplementary material for: Differential responses to group and individual emotional voices: a behavioral and ERP study
Source: Soc Cogn Affect Neurosci. 2026 Jun 3;21(1):nsag041. doi: 10.1093/scan/nsag041 (PMC13283483; doi:10.1093/scan/nsag041)
Supplement: nsag041_Supplementary_Data [file nsag041_supplementary_data.docx]

**Supplementary Material**

Table S1. Results of the descriptive statistics of behavioral data

| Stimulus type | Emotion type | **Accuracy** | | **Reaction time** | | **Emotional reaction** | |
| --- | --- | --- | --- | --- | --- | --- | --- |
|  |  | *M* | *SD* | *M* | *SD* | *M* | *SD* |
| Individual | Negative | 0.93 | 0.08 | 1259.50 | 177.01 | 2.69 | 1.13 |
|  | Neutral | 0.94 | 0.14 | 1099.20 | 189.84 | 4.41 | 0.92 |
|  | Positive | 0.96 | 0.07 | 1190.23 | 161.63 | 7.26 | 1.09 |
| Group | Negative | 0.78 | 0.20 | 1313.64 | 216.96 | 3.65 | 1.33 |
|  | Neutral | 0.96 | 0.13 | 1046.52 | 190.36 | 4.40 | 1.14 |
|  | Positive | 0.97 | 0.05 | 1144.83 | 197.56 | 7.36 | 1.18 |

Table S2. Results of the statistical analyses of behavioral data

|  | **Accuracy** | | | **Reaction time** | | | | **Emotional reaction** | | |
| --- | --- | --- | --- | --- | --- | --- | --- | --- | --- | --- |
|  | *F* | *p* | η^2^_p_ | *F* | *p* | η^2^_p_ | *F* | | *p* | η^2^_p_ |
| Stimulus type | **31.13** | **< 0.001** | **0.49** | 1.76 | 0.193 | 0.05 | **23.52** | | **< 0.001** | **0.42** |
| Emotion type | **14.70** | **< 0.001** | **0.31** | **64.23** | **< 0.001** | **0.66** | **107.45** | | **< 0.001** | **0.77** |
| Stimulus type × Emotion type | **22.72** | **< 0.001** | **0.41** | **13.68** | **< 0.001** | **0.29** | **23.70** | | **< 0.001** | **0.42** |

*Note.* Significant effects (*p* < 0.05) are highlighted in bold.

**
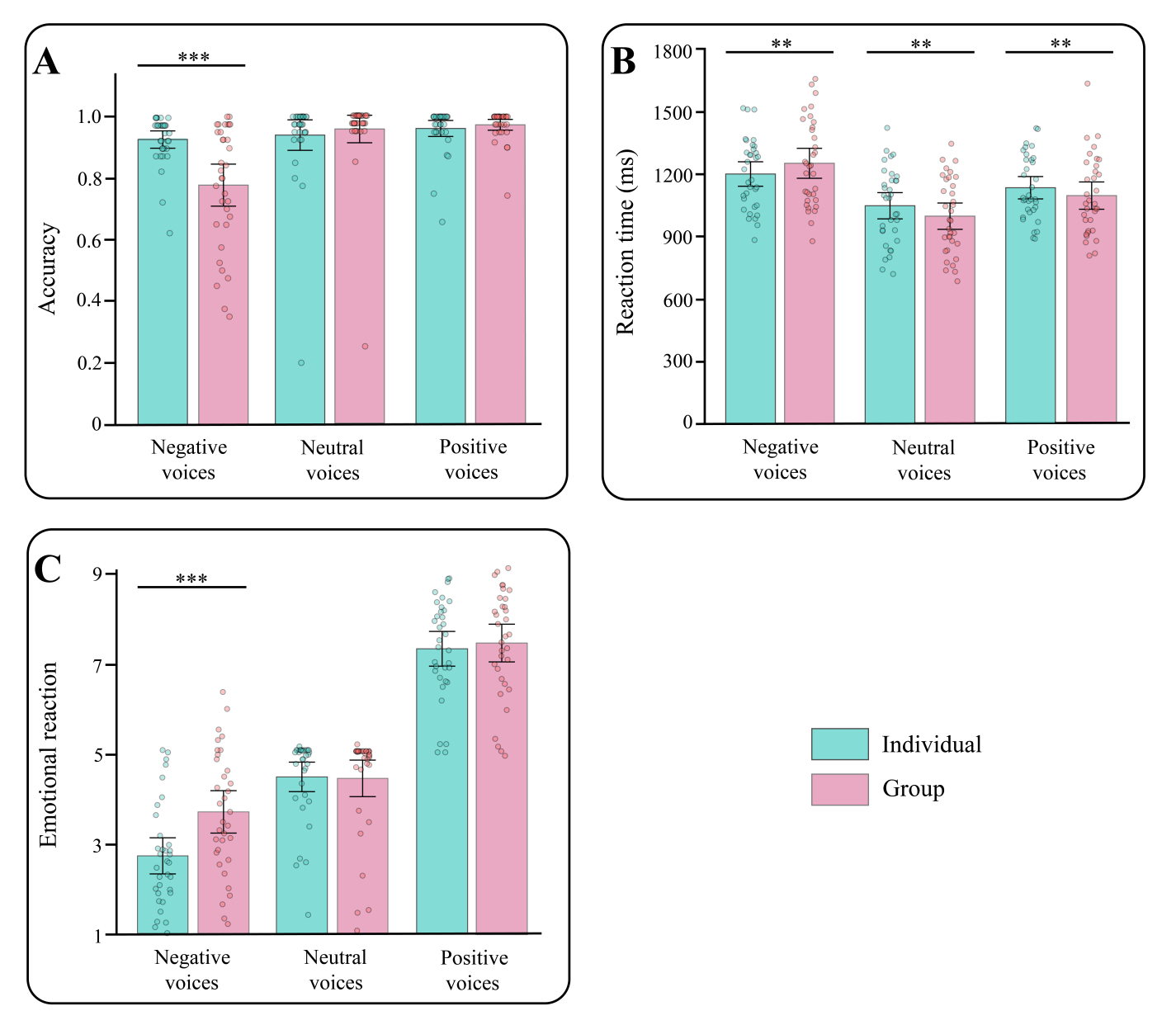
**

**Figure S1.** Behavioral results. Bar charts show behavioral responses to individual (green) and group (red) negative (left column), neutral (middle column), and positive (right column) voices. Data of accuracy, reaction time, and emotional reactions are shown in the panels A, B and C, respectively. Data in the bar charts are expressed as *mean* ± *SEM*. **: *p* < 0.01, ***: *p* < 0.001.
